# Sensors and Devices Guided by Artificial Intelligence for Personalized Pain Medicine

**DOI:** 10.34133/cbsystems.0160

**Published:** 2024-09-13

**Authors:** Yantao Xing, Kaiyuan Yang, Albert Lu, Ken Mackie, Feng Guo

**Affiliations:** ^1^ Department of Intelligent Systems Engineering, Indiana University Bloomington, Bloomington, IN 47405, USA.; ^2^ Culver Academies High School, Culver, IN 46511, USA.; ^3^ Gill Center for Biomolecular Science, Department of Psychological and Brain Sciences, Indiana University Bloomington, Bloomington, IN 47405, USA.

## Abstract

Personalized pain medicine aims to tailor pain treatment strategies for the specific needs and characteristics of an individual patient, holding the potential for improving treatment outcomes, reducing side effects, and enhancing patient satisfaction. Despite existing pain markers and treatments, challenges remain in understanding, detecting, and treating complex pain conditions. Here, we review recent engineering efforts in developing various sensors and devices for addressing challenges in the personalized treatment of pain. We summarize the basics of pain pathology and introduce various sensors and devices for pain monitoring, assessment, and relief. We also discuss advancements taking advantage of rapidly developing medical artificial intelligence (AI), such as AI-based analgesia devices, wearable sensors, and healthcare systems. We believe that these innovative technologies may lead to more precise and responsive personalized medicine, greatly improved patient quality of life, increased efficiency of medical systems, and reducing the incidence of addiction and substance use disorders.

## Introduction

Pain, a complex and subjective experience, markedly diminishes individual quality of life and imposes substantial burdens on healthcare systems [1,2]. It is the primary reason for seeking medical care, with osteoarthritis, back pain, and headaches being among the top 10 causes for patient visits, according to the Rochester Epidemiology Project (REP) medical records [3]. The Global Burden of Disease (GBD) 2010 study positioned tension-type headaches (TTHs) as the world’s second most prevalent condition (22%), trailing only dental caries and slightly ahead of migraines (15%) [4]. In the United States, the prevalence of high-impact chronic pain was 7.4% according to the National Center for Health Statistics (NCHS) reports [5]. Despite the acknowledged universality and significance of pain, its accurate assessment and effective management pose persistent difficulties [6].

Personalized medicine is increasingly valuable in pain management, enhancing treatment efficacy and addressing individual patient needs. For example, personalized medicine can leverage genetic markers and wearable devices to tailor treatments and adjust dosages in real time, ensuring more effective, safer, and timely pain management [7]. However, the gap between technological implementation and clinical application limits its wider adoption in the field. Pain biomarkers offer vital biological insights, facilitating more objective evaluations of pain severity and characteristics [8,9]. These markers, which may be specific molecules, gene expression patterns, or physiological indicators, help signal inflammation, tissue damage, or neural changes. Utilizing these biomarkers allows for more precise diagnosis and tailored treatment strategies [8,10]. However, due to the subjectivity and complexity of pain, no single biomarker can fully capture all its facets. Therefore, integrating multiple biomarkers with clinical assessment tools remains a critical goal in advancing personalized pain medicine through thorough and accurate pain evaluation [11].

Currently, pharmacological interventions remain the most common and effective method for treating pain. However, many drugs used for alleviating pain have harmful side effects. For example, opioids, despite their potent analgesic effects, are notorious for their high potential for addiction, tolerance, dependence, and risk of overdose. Nonsteroidal anti-inflammatory drugs, while effective in alleviating many kinds of pain, can cause gastrointestinal bleeding and kidney damage and increase the risk of heart disease. The side effects associated with these medications highlight the need for safer, more targeted pain management strategies that reduce dependence on traditional drugs and improve treatment outcomes [12]. Additionally, ongoing exploration of alternative, nonpharmacological therapies with fewer side effects, such as physical therapy, also holds potential to enhance pain management.

Artificial intelligence (AI) is rapidly becoming essential in personalized pain medicine [13]. Utilizing data analytics and machine learning, AI can process extensive patient data, identify pain patterns, and predict treatment outcomes. AI can also dynamically adjust treatment plans based on real-time pain changes through continuous monitoring and feedback. These advances boost treatment efficiency and reduce drug dependency side effects, significantly enhancing pain management quality. AI-guided sensors and devices are revolutionizing pain assessment and management. These advanced tools combine sensors with AI algorithms and personalized analgesia technology to monitor pain-related data in real time. They analyze physiological and behavioral indicators and provide tailored analgesic responses based on individual pain profiles, offering precise and adaptive pain treatment. For instance, wearable sensor devices can assess pain levels by monitoring heart rate, blood pressure, electromyography (EMG), and electrodermal activity (EDA) [14]. Machine learning techniques then analyze these data to identify pain patterns and tailor management strategies for individual patients [15]. Moreover, smart devices enhance pain management interactivity and personalization. Through mobile applications, patients can document pain occurrences, aiding in the adjustment of treatment plans. These devices also facilitate cognitive behavioral therapy (CBT) and psychosocial support via remote physician consultations or interactions with AI large language models (LLMs), thus addressing the emotional and psychological aspects of chronic pain management [16].

Despite the promising potential of smart devices and sensors in personalized pain medicine, challenges such as data accuracy, device reliability, privacy, security concerns, and the cost of technology need to be addressed. This article explores pain concepts, intelligent assessment, and pain relief devices, and envisages advanced pain management systems, discussing potential challenges and future innovations.

### Personalized pain medicine

**Fig. 1.**
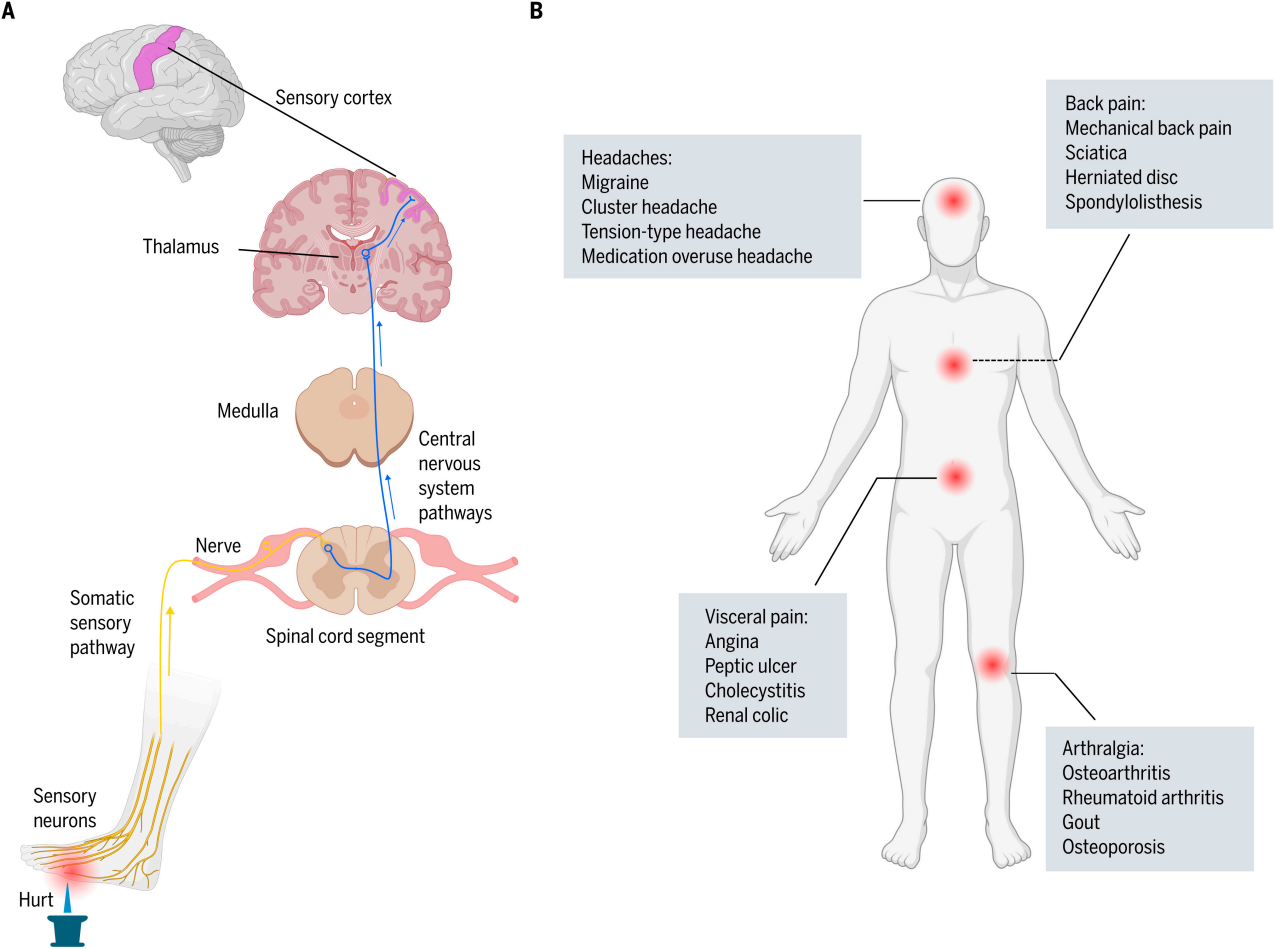
Sites and mechanisms of pain. (A) Schematics of the nociceptive/pain pathways from periphery to brain. Painful stimuli activate and depolarize the peripheral terminals of nociceptors. Action potentials are then transmitted along the afferent axons to the spinal cord. Neurotransmitters released from nociceptive neurons activate spinal neurons that send pain signals across the spinal cord and up specific fiber tracts, terminating in the medulla, midbrain, and thalamus. Projections from thalamic neurons relay these signals to regions of the cortex including the somatosensory cortex where the neural signals are interpreted as pain localized to a specific body region. (B) Pain sites and related subtypes.

Pain represents a multifaceted physiological process involving numerous cells, neurotransmitters, and pathways. Its physiological underpinnings are principally categorized into three processes: the generation, transmission, and perception of pain (Fig. 1A). The initiation of pain (nociception) emanates from the release of chemicals by damaged tissues, which activate surrounding nociceptors (“pain” receptors). Notable activators include prostaglandins, bradykinin, and serotonin. Upon activation, nerve fibers—predominantly Aδ-fibers and C-fibers—relay these signals from the affected area to the spinal cord and then to the brain. Aδ-fibers are responsible for conveying sharp, acute pain, whereas C-fibers typically transmit sensations of dull, sustained, and poorly localized pain. Upon reaching the brain, nociceptive signals are interpreted by the sensory and prefrontal cortex in particular, which culminates in the subjective sensation of pain [17,18]. Deeper brain structures, such as the hypothalamus and amygdala, play pivotal roles in modulating emotional responses to pain. Pain transcends physiological phenomena, encompassing psychological and emotional dimensions. The International Association for the Study of Pain (IASP) currently defines pain as “an unpleasant sensory and emotional experience associated with actual or potential tissue damage, or described in terms of such damage” [19]. Pain sensations can range from mild to severe and may manifest as pricking, tingling, stinging, burning, shooting, aching, or electric sensations [1].

Pain is classified using multiple methodologies (Fig. 1B). Based on duration and frequency, primary pain patterns are distinguished as follows: (a) Acute pain is characterized by sudden onset and subsequent cessation upon the resolution or treatment of its cause. This type of pain serves as a warning of potential bodily harm from injuries, diseases, overuse, or environmental stressors, thus possessing survival value and facilitating healing by alerting individuals to harmful bodily changes or teaching avoidance of harmful stimuli. Common originators of acute pain include strained muscles, fractures, dental procedures, surgeries, childbirth, infections, lacerations, and burns. (b) Episodic pain occurs sporadically and at irregular intervals. This pain type can arise unexpectedly or be triggered by known factors. Examples include painful periods, chronic migraines, and chronic medical conditions such as sickle cell anemia. (c) Chronic pain persists beyond 3 months or the anticipated healing period. In some instances, acute pain may evolve into chronic pain. Unlike acute pain, chronic pain offers minimal evolutionary advantage and may persist without an identifiable cause or continue after the resolution of an injury or known cause. Chronic pain can significantly impact mood, relationships, mobility, and the ability to perform daily tasks [20,21].

Pain can further be categorized by its source: (a) Nociceptive pain, arising from tissue damage or inflammation, varies in sensation—sharp, pricking, dull, or aching—based on the causative factor. It is also the predominant form of chronic pain. (b) Neuropathic pain, primarily resulting from nerve damage due to injury or disease, is often described as burning, tingling, shooting, or akin to electric shocks, with conditions such as diabetic neuropathy, shingles, and sciatica being common causes. (c) Nociplastic pain is caused by maladaptive changes affecting nociceptive processing and modulation without evidence of tissue or nerve damage. It involves mechanisms such as central sensitization, wind-up phenomena, glial and chronic immune system activation, altered responses to psychosocial stressors, and reduced central inhibition. Conditions like fibromyalgia, irritable bowel syndrome, and tension headaches exhibit nociplastic pain [1,22].

Chronic pain, among the types discussed, not only lacks evolutionary benefits but also imposes significant physiological and psychological burdens [23]. Many chronic pain conditions, particularly those related to cancer and spinal disorders, exhibit a mixed pain phenotype. The etiology of chronic pain is multifaceted, often encompassing factors such as patient movements and behaviors, alterations in fluid and chemical balances, lesions in muscle and visceral tissues, neurological damage, and the psychological state of the patient, necessitating multidimensional diagnosis and the formulation of comprehensive treatment plans. Consequently, the development of effective diagnosis and assessment tools for chronic pain is of paramount importance.

### Sensors and devices for pain sensing

In modern medicine, accurately monitoring and evaluating pain is critical for its optimal treatment. The integration of Internet of Things (IoT) and data-driven technologies has led to the development of devices capable of real-time precise pain monitoring, thereby enhancing personalized pain treatment. Wearable sensors play a crucial role by measuring physiological and biochemical indicators, which provide insights into individual pain response patterns [24,25]. Intelligent communication devices are vital for psychological assessment and facilitating dialogue, both of which are essential components of comprehensive and personalized pain medicine. Together, these technological advancements offer a holistic approach to pain evaluation, significantly improving the personalization and effectiveness of treatment strategies.

#### Wearable physical/chemical sensory device

The development of intelligent wearable sensors represents a significant leap forward in the monitoring and evaluation of pain responses, offering a noninvasive and portable approach for the real-time tracking and analysis of health indicators [26]. These devices, which include watches, wristbands, and patches, facilitate the continuous monitoring of physiological and biochemical markers, thereby broadening the horizons of personalized pain medicine.

**Fig. 2.**
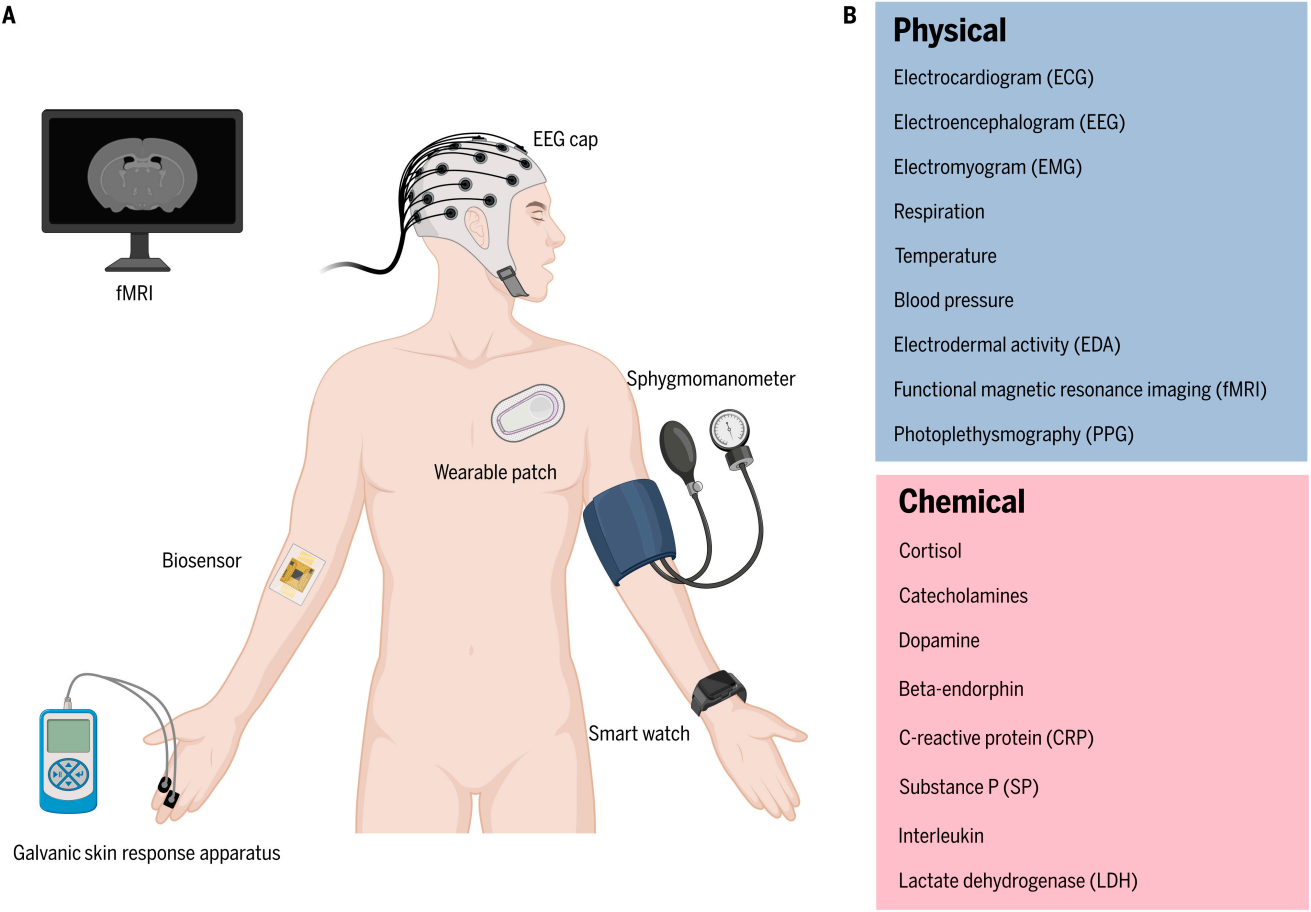
Current devices and biomarkers in pain management. (A) Common devices and sensors for pain assessment mounted on different accessories or skin. (B) Key physical and chemical biomarkers that reflect pain conditions and can be measured by current devices.

Intelligent wearable physical sensors (Fig. 2A) are capable of measuring a variety of physical parameters (Fig. 2B) pertinent to pain, such as body temperature, cardiac activity [electrocardiogram (ECG)], blood pressure, muscle activity (EMG), and brain signals [electroencephalogram (EEG)], etc. [27–29] Through the real-time tracking of these parameters, such sensors are instrumental in discerning physiological patterns associated with pain and in monitoring the evolution of pain. For instance, sensors that measure muscle bioelectrical signals can ascertain muscle strain resulting from pain and help evaluate the state and function of injured muscles by analyzing electrical activity within the muscles [30,31], while sensors measuring skin temperature can detect variations in local blood flow attributable to pain and inflammation [32] (Table 1).

**Table 1.** Potential physiological parameters for pain monitoring and assessment

Physiological data	Sensitivity	Frequency range	Amplitude range	Sampling frequency
ECG	100 μV	0.05–110 Hz	0.5–5 mV	>200 Hz
EMG	5 μV	30–300 Hz	5–1,000 μV	>500 Hz
EDA	0.01 μSiemens	0.01–1 Hz	1–20 μSiemens	>50 Hz
EEG	0.5 μV	0.5–500 Hz	1–100 μV	>1,000 Hz
PPG		0.5–5 Hz		>100 Hz
Respiration		0.1–0.5 Hz		25 Hz
Blood pressure	1 mmHg		40–300 mmHg	>0.01 Hz
Temperature	0.1 °C		35.0–42.0°C	>0.01 Hz

Complementary to physical sensors, intelligent wearable chemical sensors analyze changes in bodily chemicals or in examining trace biological samples such as sweat. These sensors are adept at monitoring bodily chemical levels, including electrolytes, metabolites, and other biomarkers, which are essential for assessing the body’s inflammatory response or biochemical condition [24,33–35] (Fig. 2B). By evaluating specific biomarkers in sweat, for example, chemical sensors can yield critical insights into an individual’s pain status, reflecting inflammation levels or other biochemically related pain processes [36].

The development of wearable sensors and devices is promising for pain assessment, but it is still challenging to fully explore their potential in clinical practice because of several possible reasons: First, the subjective nature of pain requires collecting extensive physiological and biochemical data from each individual. However, capturing an individual’s pain experience remains challenging due to variations from patient to patient especially for psychological and emotional states [37]. Moreover, the advanced algorithms and expertise required for these technologies can complicate data interpretation, potentially increasing the risk of misdiagnosis [14]. Environmental variations and the inappropriate usage of device during long-term monitoring also affect sensor accuracy, thereby limiting data interpretation. Issues such as the comfort and invasiveness of long-term wear, the high cost of devices, and a lack of standardization are significant barriers to the widespread adoption of these technologies [38] (Table 2). Beyond performance, data security deserves equal attention. With the generation and transmission of vast amounts of data, securing and protecting these data becomes another major challenge [39].

**Table 2.** Performance comparison of different pain sensors and devices

Sensing type	Signal	Sensor	Mainstream material	Advantage	Limitation
Electrical	ECG, EMG, EEG, EDA	Electrodes	Ag/AgCl [164]	High performance and stability	Low biocompatibility
			Polypyrrole (PPy) [165]	High performance	Low stability
			Carbon nanotubes (CNTs) [166]	High performance and suitable for Flexibility	Low biocompatibility
	Respiration, PPG	Piezoelectric sensors	Polyvinylidene fluoride (PVDF) [167]	High performance and suitable for Flexibility	Low piezoelectric efficiency
			Piezoelectric nanomaterials [168]	High performance	High cost
Optical	PPG	Photodiode [169]		High sensitivity and low noise	No built-in gain
		Phototransistor [170]		High sensitivity	High noise and low responding speed
Thermal	Temperature	Thermal resistance	Platinum thermal resistor [171]	High accuracy but slow	Slow responding time
		Infrared temperature sensor		Low accuracy	Quick responding time

ECG, electrocardiogram; EMG, electromyography; EEG, electroencephalogram; EDA, electrodermal activity; PPG, photoplethysmographic

Smart wearable sensors enhance the precision and immediacy of pain assessments and facilitate the real-time transmission of data to smartphones or the computers of medical professionals via wireless technology, enabling remote pain monitoring and management. Moreover, the integration of sophisticated algorithms and AI technologies allows for the comprehensive analysis of collected data, aiding in the identification of potential pain causes and trends [14,40,41]. This not only aids in the formulation of personalized pain medicine strategies but also lays a scientific foundation for the development of novel treatment modalities.

#### Imaging devices

Increased integration of imaging technology into the medical domain has provided pivotal contributions to pain assessment and management (Fig. 2A and B). This technology enables clinicians to directly visualize the internal workings of the brain and body [42–45]. Technological advancements have ushered in a range of sophisticated imaging modalities including functional magnetic resonance imaging (fMRI), which monitors changes in blood oxygen level-dependent (BOLD) signals to observe brain activity in real time without radiation exposure; positron emission tomography (PET), which offers vital insights into the body’s biochemical processes by detecting the distribution of radioactively labeled molecules; computed tomography (CT), which employs x-rays to capture images from multiple angles within the body, subsequently generating 3D images through computer processing; and ultrasound (US), which uses high-frequency sound waves to noninvasively and nonradioactively produce images of internal organs and structures. These advanced imaging technologies not only can provide in-depth insights in pain assessment but also may have some limitations in the application of personalized pain medicine. For example, the cost may be still not well acceptable, since the purchase and maintenance expenses of high-end imaging equipment may limit their dissemination in low-resource settings [46]. Moreover, imaging techniques such as PET and CT may involve radiation exposure, raising healthy concerns for certain patient populations [47]. While US is relatively safer, its image quality is significantly influenced by the operator’s technique and the patient’s positioning, affecting diagnostic accuracy [48]. The availability of these imaging technologies and the updating of equipment can lead to significant disparities in services across different regions and healthcare facilities [49].

In the age of big data, the synergy between these imaging data and data-driven algorithms has assumed an even more significant role. AI models are proficient in detecting subtle changes or anomalies within images that may indicate pain—details that might elude human observation [50,51]. Each of these imaging and analytical technologies brings distinct advantages, collectively offering potent tools for both pain science research and clinical interventions.

#### Intelligent communication devices

Intelligent communication devices, leveraging information transmission systems [52] and LLMs such as ChatGPT [53], are pioneering new pathways for pain assessment [54,55]. Psychological evaluations and conversations are integral to personalized pain medicine, aiding healthcare practitioners in pinpointing and comprehending psychological elements that might precipitate or amplify pain. Furthermore, they facilitate the provision of efficacious coping mechanisms for patients. This approach acknowledges that pain, a subjective experience, transcends physical phenomena, being significantly shaped by an individual’s emotional, psychological, and social milieu. Prompt interactions with healthcare providers can markedly assist in acquiring up-to-date insights into a patient’s pain and psychological condition, thereby enabling the tailoring of advice or personalized treatment regimens [16]. Despite recent advancements these devices are still underdeveloped for pain management. More efforts need to be made to ensure the strict confidentiality and security of patient information [56]. Additionally, although these devices offer multilingual communication flexibility through LLMs, their technical complexity can be daunting for some users, especially those less familiar with advanced technologies [57]. Furthermore, AI’s capabilities in understanding complex human emotions and psychological states are still limited and may not fully replace the role of human doctors in psychological assessments and pain management [58]. Over-reliance on technology-driven communication may overlook the importance of direct human interaction [59], which is particularly crucial when addressing pain-related psychological and emotional issues.

Engagements with AI dialogue systems are particularly beneficial for routine contexts, offering patients the flexibility to interact in different languages, while concurrently alleviating the workload of healthcare providers. Such advancements are pivotal in augmenting the efficacy of pain treatment and in stabilizing patients’ psychological states [60–62].

#### Biomarkers for pain

Effective and comprehensive pain assessment hinges on the correct integration of multiple biomarkers. Initially, establishing the objectives for a multi-marker integration model is fundamental, as this dictates the role of biomarker integration and the selection of specific biomarkers. For example, assessing pain intensity may achieve high performance using bioelectrical signals alone [63], whereas models designed to optimize pharmacological treatment plans may need to explore patient genetic diversity and a broader range of dynamic biomarker indicators [64]. Furthermore, choosing the appropriate biomarkers is key to enhancing model performance. For instance, specific inflammatory markers such as interleukins and tumor necrosis factors significantly improve the assessment of inflammatory pain [65]. Last, the utilization of complex data analysis and machine learning methods is critical to extract useful information from a wealth of biomarkers, identifying biological and behavioral patterns related to pain. For example, employing statistical methods such as multivariate regression analysis helps understand the relationships between different biomarkers and their collective impact on pain [66]. Integrating and analyzing data through algorithmic modeling and data mining techniques identifies patterns and trends in pain assessment [67].

Integrating multiple biomarkers can enhance the precision of clinical decision-making, supporting physicians in personalized pain management and thus optimizing treatment plans and improving patient quality of life. By monitoring specific biomarkers, physicians can more accurately determine which patients might benefit from nonpharmacological treatments such as physical therapy, psychological interventions, cognitive–behavioral therapy, or neuromodulation. For instance, monitoring biomarkers related to stress responses, like cortisol levels, can enable physicians to recommend specific relaxation and psychological intervention strategies that are highly effective in managing symptoms in chronic pain patients [68]. In drug development, particularly in exploring the efficacy of nonopioid pain medications, this approach offers a method to screen and optimize potential drug candidates. By measuring inflammatory biomarkers or neurotransmitters, researchers can assess the impact of new drugs on these biological processes, thereby predicting their potential efficacy in pain treatment [69]. This method can accelerate the discovery of effective medications while reducing dependence on traditional opioids. In situations where opioid use is necessary, biomarkers can help physicians precisely adjust medication dosages to minimize dependency and potential side effects. For example, certain genetic markers can predict an individual’s metabolic rate and responsiveness to opioids, allowing for personalized medication dosing to minimize side effects and optimize therapeutic efficacy [70].

However, the practical application of this approach still faces several challenges. First, the interactions between different biomarkers are complex, requiring highly precise technologies to monitor and analyze these markers simultaneously [71]. Second, significant variations in biomarker expression among individuals necessitate that assessment and treatment approaches be tailored to individual differences [72]. Lastly, translating these biomarker research findings into clinical applications requires extensive clinical trials and validation to ensure safety and effectiveness.

In summary, technological advancements in device technologies provide a comprehensive improvement toward improved pain evaluation and management, bridging physiological, biochemical, and psychological aspects. With the aid of AI technologies, these innovations promise real-time, accurate, and holistic assessments, not only deepening our understanding of pain mechanisms but also significantly improving personalized treatment strategies. This heralds a future in medical care where pain is no longer an elusive therapeutic challenge but a condition that can be quantified, understood, and effectively managed.

### Devices for pain relief

#### Implantable drug pumps

**Fig. 3.**
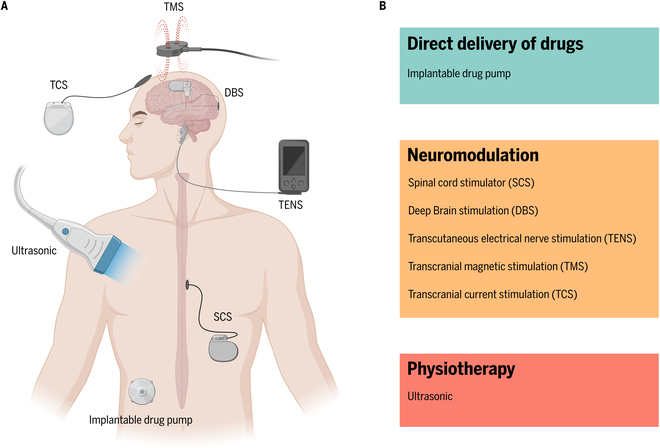
Common analgesia devices and their working principles. (A) Schematic diagram of analgesic devices used on the human body. (B) Analgesic principles of different analgesic devices.

At present, pharmacological pain relief remains the primary method for alleviating chronic pain. To better treat patients who do not adequately benefit from traditional oral or intravenous drug treatments, researchers have developed implantable drug pump technologies (Fig. 3A). According to one study [73], more than 70% of patients using implantable drug pumps reported significant pain reduction, with a corresponding decrease of greater than 50% in the need for oral pain medication. These smart implantable drug pumps can continuously and precisely deliver medication directly to the central nervous system, thus reducing the required dose of medication and the risk of systemic side effects. Comprising a medication reservoir and a micro-pump, these devices can release medication in precise doses through a small catheter implanted in the intrathecal space, based on programming determined by the needs of the patient [74] (Fig. 3B). This offers a sustained and effective management solution for difficult-to-treat chronic pain conditions such as spinal pain, cancer pain, or neuropathic pain [75,76]. Physicians can adjust the dosage and frequency of medication release, allowing for personalized treatment that minimizes side effects, enhances compliance with prescribed medical treatment, and allows for the flexible adjustment of medication release rates according to the patient’s needs [77,78].

However, the use of smart implantable drug pumps is associated with surgical and infection risks, requires regular maintenance, and incurs high costs, which may limit their widespread adoption [79]. Simultaneously, emerging technologies such as nanomedicine delivery systems, biodegradable microspheres, transdermal drug delivery systems, targeted drug delivery systems, oral controlled-release systems, and microneedle delivery systems are further enhancing the efficacy and safety of drug treatments. Preliminary studies indicate that nanomedicine delivery systems can increase treatment effectiveness by 40% to 60% while significantly reducing systemic side effects [80]. The development of these technologies not only provides more options for the treatment of chronic pain but also marks a shift toward more precise and personalized medical treatments [81].

#### Implantable analgesia devices

The spinal cord stimulator (SCS) (Fig. 3A) is an implantable device used for the treatment of chronic pain, consisting of electrodes, connection wires, and a pulse generator. Based on the “gate control theory,” the SCS interrupts the normal transmission of pain signals in the spinal cord by emitting electrical pulses, thereby reducing or blocking the sensation of pain (Fig. 3B). The SCS has been widely applied in the alleviation of symptoms in conditions such as complex regional pain syndrome (CRPS) [82], persistent lumbar back pain [83], neuropathic pain [84], angina [85], and other chronic pain conditions, demonstrating significant therapeutic effects. Studies have shown that over 50% of patients treated with SCS for CRPS report a reduction in pain by more than 50% [86], and approximately 70% of patients experience significant improvements in mobility and overall quality of life [86].

Although the SCS offers a continuous and effective solution for the management of chronic pain, it is not suitable for everyone. Before considering this treatment option, patients need to thoroughly discuss potential benefits and risks with healthcare professionals, including possible side effects and complications such as infection, device migration, or technical malfunctions [87]. Successful SCS treatment largely depends on the correct selection of patients. Generally, those who have not responded well to traditional treatments and do not have psychological health issues are more likely to benefit from SCS therapy [88]. Additionally, the successful implantation and management of the device also require a dedicated medical team with experience in this technology [89]. Follow-up studies report that many patients continue to experience reduced pain and improved quality of life after years of treatment [90], highlighting the importance of regular evaluation and timely adjustments to the device when needed.

Deep brain stimulation (DBS) (Fig. 3A) is a neurostimulation technique originally developed for treating movement disorders, which has recently been explored for chronic pain treatment. It operates by delivering electrical pulses through electrodes implanted in the brain, targeting specific areas to modulate abnormal neural signals [91] (Fig. 3B). For pain treatment, DBS focuses on regions involved in processing pain signals, such as the thalamus or certain deep brain nuclei [92]. By altering neural activity in these areas, DBS can reduce the perception of pain, offering an analgesic effect [93]. Primarily, DBS is applied in treating intractable neuropathic pain, central pain, and certain chronic pain conditions [94]. The efficacy of DBS in pain relief varies among individuals and is currently supported by a limited body of research [95]. For some patients, DBS can provide significant pain relief, especially when other treatment modalities fail [96]. However, patients must be informed about potential risks and side effects associated with DBS treatment, including infection, bleeding, device malfunction, or complexities in postoperative adjustments [97]. Given its invasive nature and potential risks, DBS is often considered a last resort treatment option, recommended after careful consideration of all factors [97].

#### Noninvasive analgesia devices

The transcutaneous electrical nerve stimulation (TENS) (Fig. 3A) device, compact and portable, relieves pain by transmitting electrical pulses to specific parts of the body through electrode pads attached to the skin. Its analgesic principle is similar to that of spinal cord stimulation (SCS), but TENS is also thought to activate opioid receptors in the central nervous system (Fig. 3B), releasing endogenous opioid-like substances such as endorphins to inhibit the transmission of pain signals, thus producing an analgesic effect [98]. TENS is widely used for managing various pain conditions, although its effectiveness varies from person to person [99]. Before choosing TENS as a pain treatment strategy, patients should discuss its appropriateness, potential effects, and correct usage with healthcare professionals to maximize treatment benefits. Moreover, while TENS is generally considered safe, it is contraindicated for those with areas of skin damage, users of pacemakers or other implanted electronic devices, and pregnant women (especially those in the early stages of pregnancy) due to the potential risk of adverse effects [100].

Transcranial magnetic stimulation (TMS) (Fig. 3A) employs an electromagnetic coil positioned on the scalp to generate transient magnetic pulses. These pulses are capable of traversing the skull to modulate the activity of neural cells within targeted brain regions (Fig. 3B). Initially aimed at treating depression and other psychiatric disorders, TMS’s role in pain treatment has been the subject of increasing research and exploration in recent years [101]. By delivering magnetic pulses at specific frequencies, TMS can activate or inhibit neurons within the brain, thereby altering pain perception and processing. It achieves pain relief by adjusting the activity in brain areas associated with pain. Primarily, TMS has been applied to chronic pain, migraines, and certain central pain syndromes. The efficacy of TMS in pain treatment varies among individuals, with some experiencing significant relief and others only mild or temporary improvement [101]. The appeal of TMS lies in its noninvasive nature, safety, and minimal risk of side effects [102]. Nevertheless, its use may be constrained by specialized equipment, the high costs of treatment, and the potential need for multiple sessions to achieve and maintain long-term benefits [103].

Transcranial current stimulation (TCS) (Fig. 3A) administers a low-level electrical current to the brain through electrodes placed on the scalp, altering neuronal excitability and thus impacting pain perception [104] (Fig. 3B). It is commonly used for treating chronic pain conditions such as chronic lower back pain, neuropathic pain, migraines, and fibromyalgia [105]. The efficacy of TCS in pain treatment varies among individuals. Some studies indicate its potential to significantly alleviate chronic pain and enhance quality of life for patients [106]. However, the effectiveness and safety of TCS require further validation through clinical research [107]. Advantages of TCS include its noninvasive nature, relative safety, ease of use, and minimal side effects [108]. Its therapeutic outcomes may be influenced by individual differences, stimulation parameters (such as current intensity, duration, and frequency), and the location of stimulation [109].

Ultrasonic technology (Fig. 3A) is widely utilized in the medical field, including the area of pain management. Therapeutic US uses mechanical vibrations of sound waves to facilitate tissue repair, alleviate pain, and generate thermal effects that increase tissue temperature and blood flow, thereby accelerating the elimination of metabolic waste and promoting the healing process [110] (Fig. 3B). As such, it is primarily employed in the treatment of conditions such as soft tissue injuries, muscle pain, and arthritis [111]. As a noninvasive approach with low risk, US therapy allows for the customization of treatment parameters based on the specific conditions and responses of patients, making it suitable for a wide range of pain conditions [112]. Additionally, interventions guided by US utilize real-time imaging to precisely direct devices to the appropriate treatment area for executing accurate pain treatment procedures like nerve blocks and joint cavity injections [113]. This ensures the precision of treatments through real-time, high-resolution imaging, reduces the risk of complications, and enhances treatment outcomes [114].

### AI in personalized pain medicine

The integration of AI has catalyzed a series of transformative technological advancements. Traditional statistical analysis methods often struggle to be effective when faced with the complexity and significant individual differences in pain biomarkers. AI technologies offer a continuous and objective method for assessing pain by analyzing a multitude of physiological and behavioral data sources, such as heart rate, EMG, and facial expressions [115]. This method significantly surpasses traditional approaches that rely on patient self-reports and subjective assessments by physicians. For instance, research has demonstrated that machine learning models can accurately predict chronic pain levels, thereby reducing dependence on subjective scales [116]. Moreover, AI-guided smart drug delivery systems are capable of autonomously adjusting the dosage of medications, such as opioids, based on real-time data. This allows for more precise pain control while simultaneously minimizing the risks associated with drug dependency and side effects [117]. AI further enhances clinical decision support systems, enabling treatment recommendations that are grounded in extensive data analyses to be more scientifically accurate and reliable [118]. Through the integration of these technologies, AI not only enhances the efficiency and effectiveness of pain management but also significantly improves the treatment experience and quality of life for patients, heralding a shift toward more personalized and technologically advanced pain treatment paradigms.

### Challenges in personalized pain medicine

#### Subjectivity and individuality of pain medicine

The subjectivity and individual variability in pain perception, influenced by a confluence of genetic, psychological, cultural, and experiential factors, present significant challenges in personalized pain medicine. Genetic research suggests that up to 60% of the variance in pain sensitivity and pharmacological responses can be attributed to genetic factors [119]. Similarly, psychological states and cultural backgrounds markedly influence pain experience and its management, complicating the development of universally effective pain assessment and analgesic devices [120]. These devices necessitate sophisticated algorithms capable of quantifying subjective pain experiences and must be versatile enough to cater to diverse pain types and individual patient needs through the integration of multifaceted data sources and customizable treatment modalities. Additionally, the imperative for user-friendliness, portability, and the capability for continuous patient monitoring underscores the need for devices that can dynamically adjust treatment parameters in response to real-time feedback [121]. Furthermore, devices must be assured of safety and efficacy; particularly in light of individual differences, this requires thorough clinical validation in appropriately selected patient populations [122]. This rigorous process is indispensable for the advancement of more personalized, adaptable, and holistic personalized pain medicine strategies [123].

#### Unequal distribution of medical resources

The unequal distribution of medical resources significantly impacts pain management. This is especially evident in the disparity between high-income countries and urban areas versus low-income countries and remote regions. The World Health Organization (WHO) reports that high-income countries consume the majority of the world’s opioid medication, despite representing only a small fraction of the global population [124]. Additionally, data from the World Bank and the WHO indicate that the density of medical personnel in some low-income countries falls well below the global average, underscoring the inequality in human resource distribution [125]. This uneven allocation of resources leads to global inequalities in the accessibility and quality of health services, particularly in pain management, where many patients struggle to access effective treatment and management services, exacerbating global health disparities [126]. The high cost of medical devices, especially those designed for personalized pain medicine, further limits accessibility in lower-income regions and countries [127]. Consequently, there is an urgent need for the development and manufacture of cost-effective, easy-to-operate, and low-maintenance pain management devices. These devices must also be adaptable to varying medical environments and infrastructure limitations, addressing the pressing challenge of bridging the gap in global pain management resources [128].

#### Substance abuse and addiction

Substance abuse, especially the overuse or inappropriate use of opioids in the field of pain treatment, poses a significant challenge, severely threatening individual health and evolving into a global public health crisis. Data from the Centers for Disease Control and Prevention (CDC) reveal that in 2022, approximately 82,000 Americans died from opioid overdoses, accounting for nearly 70% of all drug overdose deaths [129]. Furthermore, the WHO estimates that around 27 million people worldwide suffer from substance use disorders due to nonmedical use of substances, highlighting the dominant role of opioids in drug-related deaths and the substantial burden on society and the economy of their misuse [130]. The root causes of substance abuse include excessive prescribing, inadequate regulation, and underestimation of drug addiction potential [131]. In the face of the escalating issue of opioid dependence and abuse, the development of nonpharmacological pain treatment devices becomes particularly crucial [132]. The challenges lie not only in proving their efficacy and safety through clinical trials but also in ensuring patient acceptance of these alternative therapies and their ease of use in practice [133]. Additionally, as medical devices play an increasingly important role in pain treatment, especially when using drug pumps or other drug delivery systems, the integration of overdose detection features is essential to timely identify the potential dangers of using high-risk drugs such as opioids [134]. Thus, more efforts will be required to develop the potential of nonpharmacological therapies, such as magnetic neural stimulation, acoustic neuromodulation, and new medication, which may bring new insights to manage overdosed events and reduce dependency on abused substances such as opioids.

### Future development for pain medicine

#### AI-based analgesia devices

**Fig. 4.**
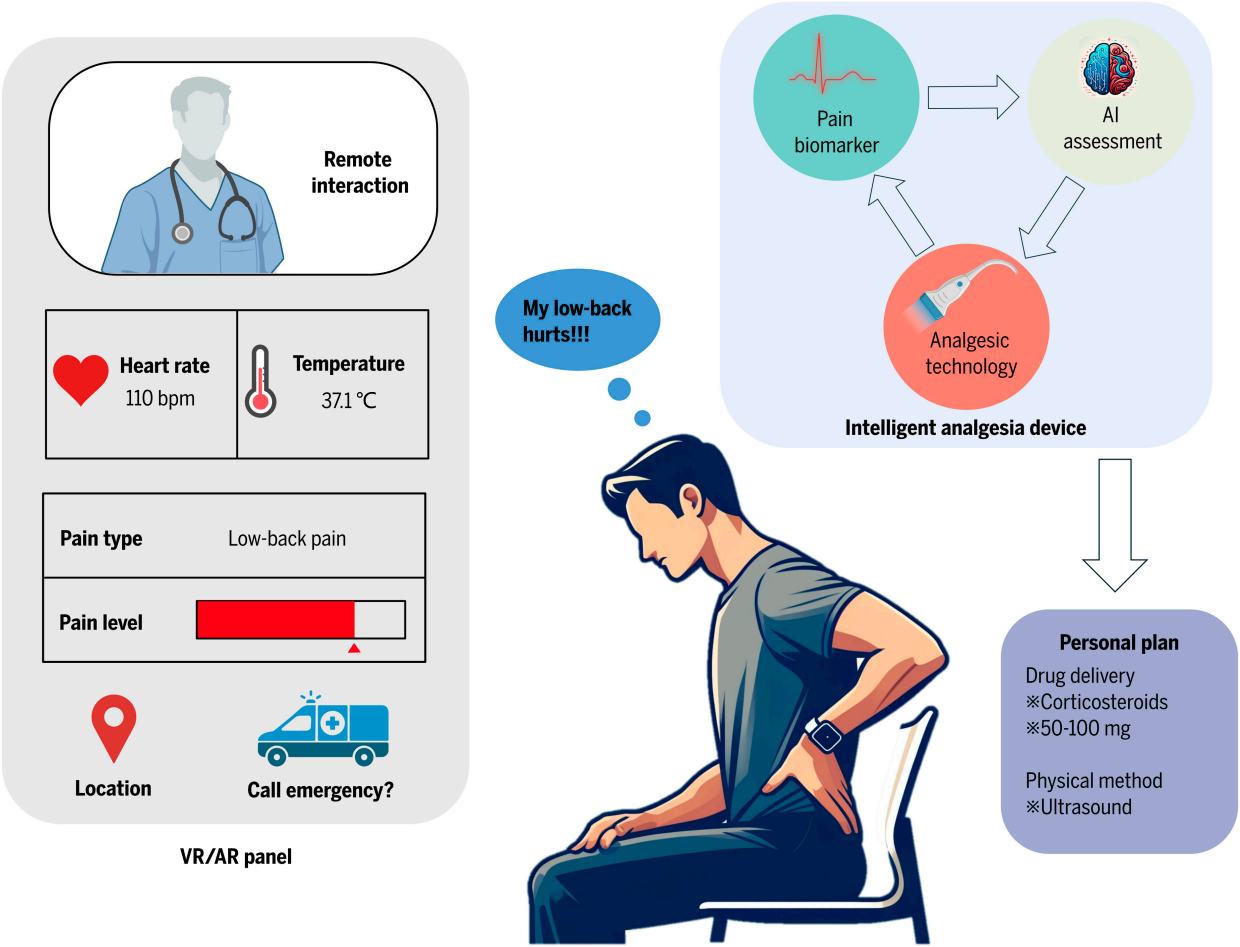
Future technologies and systems for personalized pain medicine. A patient experiencing lower back pain is wearing a smart wearable device integrated with sensors, analgesic technology, and AI. This AI-driven wearable detects the patient’s pain signals and formulates a personalized pain medicine plan to alleviate their discomfort. Additionally, the device continuously collects and analyzes the patient’s vital signs and displays them on a VR/AR panel. This helps assess the effectiveness of the pain management plan and monitors for potential issues such as medication overdose. When necessary, the patient can engage in remote interactions with healthcare providers via VR/AR technology to receive professional advice and educational support.

The effectiveness of analgesia devices varies from person to person, primarily because they require the setting of individualized parameters to be effective. Analyzing a large amount of patient data, including pain levels, treatment responses, and patient preferences, is necessary to create personalized treatment plans [135]. This requires frequent interaction between patients, doctors, and experienced medical staff [136]. AI shows tremendous potential in developing personalized pain relief plans due to its ability to process vast amounts of multimodal data and its strong learning capabilities [40]. Based on the collection of patient data from various dimensions such as physiological data, treatment data, and behavioral data, we can develop different AI models to control pain relief devices, enabling them to serve more effectively in pain management (Table 3). Intelligent devices can not only set individual treatment parameters by analyzing a patient’s medical history, type of pain, and sources of pain but also adjust the dosage of medication or the intensity and frequency of electrical stimulation based on real-time feedback from the patient, achieving precise treatment [137]. However, faced with challenges such as data privacy, algorithm transparency, and generalization capabilities, future research will need to further optimize the application of AI in personalized pain medicine with the support of technological advances and ethical and legal frameworks, providing safe and effective personalized treatment solutions for a broader patient population [138,139] (Fig. 4).

**Table 3.** Potential datasets for various pain-related operations

Categories	Details of datasets	Various pain-related operations
Basic information	• Demographic data: age, gender, weight, height, race. • Medical history: previous medical history, surgical history, allergy history, family medical history.	• Pain prediction model • Individualized dosing regimen • Pain management decision support
Pain-related data	• Pain score: the pain level reported by the patient [e.g., visual analog scale (VAS) and numerical rating scale (NRS)]. • Pain location and type: the specific location and type of pain.	• Pain prediction model • Pain assessment system • Individualized dosi ng regimen • Pain management decision support
Physiological data	• Vital signs: heart rate, blood pressure, respiratory rate, body temperature. • Dynamic monitoring data: real-time physiological data obtained through wearable devices, such as ECG, blood oxygen saturation (SpO_2_), EDA, EMG.	• Pain prediction model • Pain assessment system • Individualized dosing regimen • Pain management decision • Pain monitoring system • Pain relief device control
Laboratory test data	• Blood indicators: blood routine, biochemical indicators. • Imaging data: X-rays, CT, MRI, PET and other imaging data.	• Pain prediction model • Pain assessment system • Individualized dosing regimen • Pain management decision • Pain monitoring system
Treatment data	• Medication records: types of analgesics currently and in the past, dosage, route of administration, time of administration. • Therapeutic effect: evaluation of efficacy, side effects, adverse reactions after medication.	• Individualized dosing regimen • Pain monitoring system • Pain relief device control
Behavioral and environmental data	• Lifestyle: eating habits, smoking and drinking, exercise. • Psychological state: assessment of psychological factors such as anxiety and depression.	• Pain prediction model • Pain assessment system • Pain management decision • Pain monitoring system
Subjective data	• Patient feedback: Patients’ subjective evaluation and feedback on pain management programs. • Quality of life assessment: Patients’ self-assessed quality of life and functional status such as SF-36, EORTC QLQ-C30.	• Pain assessment system • Individualized dosing regimen • Pain management decision

#### Continuous monitoring and management of pain

With the advancement of AI technology and wearable devices, the degree of intelligence in pain monitoring and management systems has significantly increased. Current research demonstrated the effectiveness of using wearable devices to monitor heart rate, activity levels, and sleep quality in pain management [140] (Fig. 4). These devices, using real-time tracking of physiological indicators and pain conditions combined with AI algorithms to analyze data, may allow for a deeper understanding of patients’ health patterns and pain triggers [141]. The integration of microfluidic components [142–144] and point-of-care of diagnostic sensors may also further enable the real-time and in situ detection of biological pain markers [142,145,146] for further advancing the continuous monitoring and management of pain for clinical and daily life conditions [147–149]. Besides, AI can accurately predict potential triggers of pain onset, enabling patients to take preventive measures in advance [40,150]. Further studies may focus on how AI could automatically adjust the settings of pain relief devices, such as drug pumps or electrical stimulators, to modulate treatment in real time, thereby greatly enhancing the proactivity and personalization of pain management [151]. Regarding the improvement of treatment efficiency and patients’ quality of life, although specific figures depend on the design and results of each study, it is generally expected that AI’s intervention in pain management can significantly accelerate treatment responses and reduce the frequency and intensity of pain episodes, thus improving patients’ overall quality of life.

#### Virtual reality- and augmented reality-based pain treatment

AI-enhanced virtual reality (VR) and augmented reality (AR) technologies are revolutionizing the field of pain treatment by effectively distracting patients through personalized virtual environments or games, thereby alleviating pain sensations. Research has shown that patients engaged in VR environments experience significantly lower pain scores compared to traditional pain treatment methods, with notable improvements in engagement and satisfaction levels [152]. This indicates the tremendous potential of these innovations for future medical advancements (Fig. 4). By integrating AI technology, these virtual treatment programs not only become more engaging and targeted but also provide real-time monitoring of patient responses, offering essential feedback that aids healthcare professionals in optimizing personalized pain medicine strategies [153] and significantly improves the patient’s therapeutic experience. Future research will focus on quantifying the specific benefits of AI-enhanced VR/AR in personalized pain medicine, identifying the best application scenarios, and exploring their long-term impact on patient well-being [154]. Furthermore, a deeper investigation into the mechanisms by which these technologies reduce pain could lead to further advancements in treatment methods [154]. As technology progresses, these applications are expected to become more complex and customized, offering more effective pain management solutions to patients worldwide.

#### Patient education and support

The subjective experience of pain is a key factor in formulating treatment strategies (Fig. 4). While traditional interactions between doctors and patients play a crucial role in understanding and treating pain, this model has evident limitations in resource allocation, especially when doctors need to spend a significant amount of time in face-to-face communication to deeply understand a patient’s pain condition and history [155]. This not only increases the workload for healthcare professionals but also might lead to the overutilization of resources in an already strained medical system [156]. Therefore, exploring methods that can effectively manage pain while optimizing resource use is particularly important. As a solution to this challenge, AI-driven medical chatbots, providing 24-hour support and instant feedback, have already significantly improved the pain management experience for patients [157]. For instance, Woebot [158], an AI-driven chatbot initially developed for mental health support, has been extended to include applications in other medical domains such as chronic pain management. Through regular interactions, users can obtain emotional support and behavioral health recommendations, assisting them in managing psychological states associated with their pain. Besides, virtual assistants offer personalized tracking and management suggestions based on the specific conditions of patients, helping them control pain more effectively and engage in self-management [157]. As an intelligent health assessment tool, Ada enables users to enter their symptoms, providing analyses and suggestions for potential health issues [159]. This application can be effectively utilized for pain management, as it gathers detailed information about the user’s pain symptoms to provide personalized guidance and educational resources. These technologies can reduce the workload on healthcare personnel and ensure the rational allocation of resources [160].

#### Global cooperation and resource optimization

Fostering international cooperation and resource sharing is crucial on a global scale, particularly in the field of pain management. Partnerships between high-income and low-income countries can be established through the sharing of medical resources, technology, and knowledge. International organizations, such as the WHO, play a pivotal role in promoting the development and implementation of global pain management standards [161]. Additionally, financial support from international financial institutions and development banks is vital for developing pain management infrastructure and technology in low-income countries [162]. This support also aids in the local production of medical devices and pharmaceuticals, reducing reliance on imports. Education and training are key to enhancing pain management capabilities, with targeted educational and training programs for healthcare professionals in low-income countries, and the use of online courses and remote education resources significantly improving local healthcare workers’ understanding and application of advanced pain management strategies. This comprehensive international effort not only narrows the gap in medical resources between countries but also enhances the overall level of and efficiency in global pain management.

Although AI has shown tremendous potential in personalized pain medicine, it should be seen as a supplementary tool to traditional medical services, not a replacement. AI technology should be combined with the professional knowledge and human care of healthcare professionals to ensure that patients receive the best possible pain management experience [163]. Future research on the application of AI in pain management may focus on evaluating its effectiveness in reducing pain, improving patient satisfaction, and lowering medical costs. Additionally, studies will explore how to further enhance the accuracy and reliability of AI technology to better serve personalized pain medicine by refining algorithms and integrating more diverse data sources, thereby increasing its utility in clinical settings.

## Conclusion

In summary, this review critically examines the role of sensors and devices guided by AI in the field of personalized pain medicine, highlighting their transformative impact on treatment outcomes and patient quality of life. The potential of these intelligent sensors and devices to provide real-time, accurate pain assessment and responsive treatment options marks a pivotal shift toward more dynamic and patient-specific approaches. However, the adoption of these sophisticated technologies is accompanied by substantial technical, ethical, and practical challenges, notably including the critical need to ensure data privacy, manage the complexity of integrating AI systems, and enhance interoperability with existing medical infrastructures. Future research must address these challenges head-on, refining algorithms and enhancing system interoperability to foster broader adoption. As we look to the future, the field of pain medicine is poised for a paradigm shift, with AI-driven technologies at the forefront of this transformation. It is imperative that future studies not only continue to advance the technological capabilities but also rigorously evaluate their impact across diverse patient populations and pain conditions. Additionally, there is a need to explore the ethical dimensions of AI in pain management, ensuring that these innovations contribute positively to patient care without exacerbating existing disparities. This review serves as a call to action for the multidisciplinary collaboration necessary to harness the full potential of sensors and devices guided by AI in revolutionizing pain management. The integration of these technologies into clinical practice promises not only enhanced patient outcomes but also a more nuanced understanding of pain mechanisms, ultimately leading to more effective and personalized treatment strategies.

## Data Availability

Data sharing is not applicable to this article, as no new data were created or analyzed in this study.

## References

[1] Cohen SP, Vase L, Hooten WM. Chronic pain: An update on burden, best practices, and new advances. Lancet. 2021;397(10289):2082–2097. 34062143 10.1016/S0140-6736(21)00393-7

[2] Smith BH, Elliott AM, Chambers WA, Smith WC, Hannaford PC, Penny K. The impact of chronic pain in the community. Fam Pract. 2001;18(3):292–299. 11356737 10.1093/fampra/18.3.292

[3] Sauver JLS, Warner DO, Yawn BP, Jacobson DJ, ME MG, Pankratz JJ, Melon LJ III, Roger VL, Ebbert JO, Rocca WA. Why patients visit their doctors: Assessing the most prevalent conditions in a defined American population. Mayo Clin Proc. 2013;88(1):56–67. 23274019 10.1016/j.mayocp.2012.08.020 PMC3564521

[4] Vos T, Flaxman AD, Naghavi M, Lozano R, Michaud C, Ezzati M, Shibuya K, Salomon JA, Abdalla S, Aboyans V, et al. Years lived with disability (YLDs) for 1160 sequelae of 289 diseases and injuries 1990–2010: A systematic analysis for the Global Burden of Disease Study 2010. Lancet. 2012;380(9859):2163–2196. 23245607 10.1016/S0140-6736(12)61729-2 PMC6350784

[5] Zelaya CE, Dahlhammer JM, Lucas JW, Connor EM. Chronic pain and high-impact chronic pain among US adults, 2019. *NCHS Data Brief*. 2020. p. 1–8. 33151145

[6] Gatchel RJ, McGeary DD, McGeary CA, Lippe B. Interdisciplinary chronic pain management: Past, present, and future. Am Psychol. 2014;69(2):119–130. 24547798 10.1037/a0035514

[7] Bruehl S. Personalized pain medicine: Pipe dream or reality? Anesthesiology. 2015;122(5):967–968. 25751235 10.1097/ALN.0000000000000638 PMC4439303

[8] Mouraux A, Iannetti GD. The search for pain biomarkers in the human brain. Brain. 2018;141(12):3290–3307. 30462175 10.1093/brain/awy281 PMC6262221

[9] Cai H, Ao Z, Tian C, Wu Z, Kaurich C, Chen Z, Gu M, Hohmann AG, Mackie K, Guo F. Engineering human spinal microphysiological systems to model opioid-induced tolerance. Bioact Mat. 2023;22:482–490. 10.1016/j.bioactmat.2022.10.007 PMC9618681 36330161

[10] Ao Z, Cai H, Wu Z, Krzesniak J, Tian C, Lai YY, Mackie K, Guo F. Human spinal organoid-on-a-chip to model nociceptive circuitry for pain therapeutics discovery. Anal Chem. 2021;94(2):1365–1372. 34928595 10.1021/acs.analchem.1c04641 PMC11483356

[11] Davis KD, Aghaeepour N, Ahn AH, Angst MS, Borsook D, Brenton A, Burczynski ME, Crean C, Edwards R, Gaudilliere B, et al. Discovery and validation of biomarkers to aid the development of safe and effective pain therapeutics: Challenges and opportunities. Nat Rev Neurol. 2020;16(7):381–400. 32541893 10.1038/s41582-020-0362-2 PMC7326705

[12] Xu J, Cai H, Wu Z, Li X, Tian C, Ao Z, Niu VC, Xiao X, Jiang L, Khodoun M, et al. Acoustic metamaterials-driven transdermal drug delivery for rapid and on-demand management of acute disease. Nat Commun. 2023;14(1):869. 36797284 10.1038/s41467-023-36581-2 PMC9935629

[13] Lötsch J, Ultsch A. Machine learning in pain research. Pain. 2018;159(4):623–630. 29194126 10.1097/j.pain.0000000000001118 PMC5895117

[14] Chen J, Abbod M, Shieh JS. Pain and stress detection using wearable sensors and devices—A review. Sensors. 2021;21(4):1030. 33546235 10.3390/s21041030 PMC7913347

[15] Matsangidou M, Liampas A, Pittara M, Pattichi CS, Zis P. Machine learning in pain medicine: An up-to-date systematic review. Pain Ther. 2021;10(2):1067–1087. 34568998 10.1007/s40122-021-00324-2 PMC8586126

[16] Eccleston C, Morley SJ, Williams ACC. Psychological approaches to chronic pain management: Evidence and challenges. Br J Anaesth. 2013;111(1):59–63. 23794646 10.1093/bja/aet207

[17] Stucky CL, Gold MS, Zhang X. Mechanisms of pain. Proc Natl Acad Sci USA. 2001;98(21):11845–11846. 11562504 10.1073/pnas.211373398 PMC59728

[18] Basbaum AI, Bautista DM, Scherrer G, Julius D. Cellular and molecular mechanisms of pain. Cell. 2009;139(2):267–284. 19837031 10.1016/j.cell.2009.09.028 PMC2852643

[19] Raja SN, Carr DB, Cohen M, Finnerup NB, Flor H, Gibson S, Keefe FJ, Mogil JS, Ringkamp M, Sluka KA, et al. The revised international association for the study of pain definition of pain: Concepts, challenges, and compromises. Pain (2020);161(9):1976–1982. 32694387 10.1097/j.pain.0000000000001939 PMC7680716

[20] Pain; https://www.ninds.nih.gov/health-information/disorders/pain.

[21] Ashburn MA, Staats PS. Management of chronic pain. Lancet. 1999;353(9167):1865–1869. 10359427 10.1016/S0140-6736(99)04088-X

[22] Jayakar S, Shim J, Jo S, Bean BP, Singeç I, Woolf CJ. Developing nociceptor-selective treatments for acute and chronic pain. Sci Transl Med. 2021;13(619):eabj9837. 34757806 10.1126/scitranslmed.abj9837 PMC9964063

[23] Clauw DJ, Essex MN, Pitman V, Jones KD. Reframing chronic pain as a disease, not a symptom: Rationale and implications for pain management. Postgrad Med. 2019;131(3):185–198. 30700198 10.1080/00325481.2019.1574403

[24] Kim J, Campbell AS, Esteban-Fernández de Ávila B, Wang J. Wearable biosensors for healthcare monitoring. Nat Biotechnol. 2019;37(4):389–406. 30804534 10.1038/s41587-019-0045-y PMC8183422

[25] Ray TR, Choi J, Bandodkar AJ, Krishnan S, Gutruf P, Tian L, Ghaffari R, Rogers JA. Bio-integrated wearable systems: A comprehensive review. Chem Rev. 2019;119(8):5461–5533. 30689360 10.1021/acs.chemrev.8b00573

[26] Sim I. Mobile devices and health. N Engl J Med. 2019;381(10):956–968. 31483966 10.1056/NEJMra1806949

[27] Khan Y, Ostfeld AE, Lochner CM, Pierre A, Arias AC. Monitoring of vital signs with flexible and wearable medical devices. Adv Mater. 2016;28(22):4373–4395. 26867696 10.1002/adma.201504366

[28] Swaroop KN, Chandu K, Gorrepotu R, Deb S. A health monitoring system for vital signs using IoT. Internet Things. 2019;5:116–129.

[29] Xing Y, Zhang Y, Xiao Z, Yang C, Li J, Cui C, Wang J, Chen H, Li J, Liu C. An artifact-resistant feature SKNAER for quantifying the burst of skin sympathetic nerve activity signal. Biosensors. 2022;12(5):355. 35624656 10.3390/bios12050355 PMC9138869

[30] Flaxman TE, Alkjaer T, Smale KB, Simonse EB, Krogsgaard MR, Benoit DL. Differences in EMG–moment relationships between ACL–injured and uninjured adults during a weight-bearing multidirectional force control task. J Orthop Res. 2019;37(1):113–123. 30259562 10.1002/jor.24145

[31] Xing Y, Zhang Y, Yang C, Li J, Li Y, Cui C, Li J, Cheng H, Fang Y, Cai C, et al. Design and evaluation of an autonomic nerve monitoring system based on skin sympathetic nerve activity. Biomed Signal Process Control. 2022;76:103681.

[32] Ramirez-GarciaLuna JL, Bartlett R, Arriaga-Caballero JE, Fraser RDJ, Saiko G. Infrared thermography in wound care, surgery, and sports medicine: A review. Front Physiol. 2022;13:838528. 35309080 10.3389/fphys.2022.838528 PMC8928271

[33] Teymourian H, Barfidokht A, Wang J. Electrochemical glucose sensors in diabetes management: An updated review (2010–2020). Chem Soc Rev. 2020;49(21):7671–7709. 33020790 10.1039/d0cs00304b

[34] Sempionatto JR, Lasalde-Ramírez JA, Mahato K, Wang J, Gao W. Wearable chemical sensors for biomarker discovery in the omics era. Nat Rev Chem. 2022;6(12):899–915. 37117704 10.1038/s41570-022-00439-w PMC9666953

[35] Yang DS, Ghaffari R, Rogers JA. Sweat as a diagnostic biofluid. Science. 2023;379(6634):760–761. 36821680 10.1126/science.abq5916

[36] Ghaffari R, Yang DS, Kim J, Mansour A, Wright JA Jr, Model JB, Wright DE, Rogers JA, Ray TR. State of sweat: Emerging wearable systems for real-time, noninvasive sweat sensing and analytics. ACS Sens. 2021;6(8):2787–2801. 34351759 10.1021/acssensors.1c01133 PMC9108092

[37] Bushnell MC, Čeko M, Low LA. Cognitive and emotional control of pain and its disruption in chronic pain. Nat Rev Neurosci. 2013;14(7):502–511. 23719569 10.1038/nrn3516 PMC4465351

[38] Mirjalali S, Peng S, Fang Z, Wang CH, Wu S. Wearable sensors for remote health monitoring: Potential applications for early diagnosis of Covid-19. Adv Mater Technol. 2022;7(1):2100545. 34901382 10.1002/admt.202100545 PMC8646515

[39] Filkins BL, Kim JY, Roberts B, Armstrong W, Miller MA, Hultner ML, Castillo AP, Ducom J-C, Topol EJ, Steinhubl SR. Privacy and security in the era of digital health: What should translational researchers know and do about it? Am J Transl Res. 2016;8(3):1560–1580. 27186282 PMC4859641

[40] Nagireddi JN, Vyas AK, Sanapati MR, Soin A, Manchikanti L. The analysis of pain research through the lens of artificial intelligence and machine learning. Pain Physician. 2022;25(2):E211. 35322975

[41] Siddiqui SA, Zhang Y, Lloret J, Song H, Obradovic Z. Pain-free blood glucose monitoring using wearable sensors: Recent advancements and future prospects. IEEE Rev Biomed Eng. 2018;11:21–35. 29993663 10.1109/RBME.2018.2822301

[42] Chou R, Qaseem A, Owens DK, Shekelle P. Clinical Guidelines Committee of the American College of Physicians. Diagnostic imaging for low back pain: Advice for high-value health care from the American College of Physicians. Ann Intern Med. 2011;154(3):181–189. 21282698 10.7326/0003-4819-154-3-201102010-00008

[43] Wager TD, Atlas LY, Lindquist MA, Roy M, Woo CW, Kross E. An fMRI-based neurologic signature of physical pain. N Engl J Med. 2013;368(15):1388–1397. 23574118 10.1056/NEJMoa1204471 PMC3691100

[44] Chapin H, Bagarinao E, Mackey S. Real-time fMRI applied to pain management. Neurosci Lett. 2012;520(2):174–181. 22414861 10.1016/j.neulet.2012.02.076 PMC3377818

[45] Hoffmann U, Truong QA, Schoenfeld DA, Chou ET, Woodard PK, Nagurney JT, Pope JH, Hauser TH, White CS, Weiner SG, et al. Coronary CT angiography versus standard evaluation in acute chest pain. N Engl J Med. 2012;367(4):299–308. 22830462 10.1056/NEJMoa1201161 PMC3662217

[46] Grisi G, Stacul F, Cuttin R, Rimondini A, Meduri S, Dalla Palma L. Cost analysis of different protocols for imaging a patient with acute flank pain. Eur Radiol. 2000;10(10):1620–1627. 11044936 10.1007/s003300000549

[47] Baker LC, Atlas SW, Afendulis CC. Expanded use of imaging technology and the challenge of measuring value. Health Aff. 2008;27(6):1467–1478. 10.1377/hlthaff.27.6.1467 18997202

[48] Gofeld M. Ultrasonography in pain medicine: A critical review. Pain Pract. 2008;8(4):226–240. 18513228 10.1111/j.1533-2500.2008.00215.x

[49] Waite S, Scott J, Colombo D. Narrowing the gap: Imaging disparities in radiology. Radiology. 2021;299(1):27–35. 33560191 10.1148/radiol.2021203742

[50] Piri R, Nøddeskou-Fink AH, Gerke O, Larsson M, Edenbrandt L, Enqvist O, Høilund-Carlsen PF, Stochkendahl MJ. PET/CT imaging of spinal inflammation and microcalcification in patients with low back pain: A pilot study on the quantification by artificial intelligence-based segmentation. Clin Physiol Funct Imaging. 2022;42(4):225–232. 35319166 10.1111/cpf.12751 PMC9322590

[51] Lamm C, Decety J, Singer T. Meta-analytic evidence for common and distinct neural networks associated with directly experienced pain and empathy for pain. NeuroImage. 2011;54(3):2492–2502. 20946964 10.1016/j.neuroimage.2010.10.014

[52] Kakria P, Tripathi NK, Kitipawang P. A real-time health monitoring system for remote cardiac patients using smartphone and wearable sensors. Int J Telemed Appl. 2015;2015:373474. 26788055 10.1155/2015/373474 PMC4692989

[53] Haupt CE, Marks M. AI-generated medical advice—GPT and beyond. JAMA. 2023;329(16):1349–1350. 36972070 10.1001/jama.2023.5321

[54] Eccleston C, Blyth FM, Dear BF, Fisher EA, Keefe FJ, Lynch ME, Palermo TM, Reid MC, Williams ACC. Managing patients with chronic pain during the COVID-19 outbreak: Considerations for the rapid introduction of remotely supported (eHealth) pain management services. Pain. 2020;161(5):889–893. 32251203 10.1097/j.pain.0000000000001885 PMC7172975

[55] Yang G, Jiang M, Ouyang W, Ji G, Xie H, Rahmani AM, Liljeberg P, Tenhunen H. IoT-based remote pain monitoring system: From device to cloud platform. IEEE J Biomed Health Inform. 2017;22(6):1711–1719. 29990259 10.1109/JBHI.2017.2776351

[56] Coiera E. *Guide to health informatics*. Boca Raton: CRC Press; 2015.

[57] Kocaballi AB, Berkovsky S, Quiroz JC, Laranjo L, Tong HL, Rezazadegan D, Briatore A, Coiera E. The personalization of conversational agents in health care: Systematic review. J Med Internet Res. 2019;21(11):e15360. 31697237 10.2196/15360 PMC6873147

[58] Doraiswamy PM, Blease C, Bodner K. Artificial intelligence and the future of psychiatry: Insights from a global physician survey. Artif Intell Med. 2020;102:101753. 31980092 10.1016/j.artmed.2019.101753

[59] Vellido A. The importance of interpretability and visualization in machine learning for applications in medicine and health care. Neural Comput Appl. 2020;32:18069–18083.

[60] Wang DQ, Feng LY, Ye JG, Zou JG, Zheng YF. Accelerating the integration of ChatGPT and other large-scale AI models into biomedical research and healthcare. MedComm. 2023;2:e43.

[61] Li J, Dada A, Puladi B, Kleesiek J, Egger J. ChatGPT in healthcare: A taxonomy and systematic review. Comput Methods Prog Biomed. 2024;245:108013. 10.1016/j.cmpb.2024.108013 38262126

[62] Tustumi F, Andreollo NA, Aguilar-Nascimento JE. Future of the language models in healthcare: The role of ChatGPT. Arq Bras Cir Dig. 2023;36(e1727). 10.1590/0102-672020230002e1727 PMC10168663 37162073

[63] Kong Y, Posada-Quintero HF, Chon KH. Sensitive physiological indices of pain based on differential characteristics of electrodermal activity. IEEE Trans Biomed Eng. 2021;68(10):3122–3130. 33705307 10.1109/TBME.2021.3065218 PMC8483589

[64] Romm EL, Tsigelny IF. Artificial intelligence in drug treatment. Annu Rev Pharmacol Toxicol. 2020;60:353–369. 31348869 10.1146/annurev-pharmtox-010919-023746

[65] Muley MM, Krustev E, McDougall JJ. Preclinical assessment of inflammatory pain. CNS Neurosci Ther. 2016;22(2):88–101. 26663896 10.1111/cns.12486 PMC6492823

[66] Tomás-Rodríguez MI, Palazón-Bru A, Martínez-St John DRJ, Navarro-Cremades F, Toledo-Marhuenda JV, Gil-Guillén VF. Factors associated with increased pain in primary dysmenorrhea: Analysis using a multivariate ordered logistic regression model. J Pediatr Adolesc Gynecol. 2017;30(2):199–202. 27693647 10.1016/j.jpag.2016.09.007

[67] Rahman QA, Janmohamed T, Pirbaglou M, Ritvo P, Heffernan JM, Clarke H, Katz J. Patterns of user engagement with the mobile app, Manage My Pain: Results of a data mining investigation. JMIR Mhealth Uhealth. 2017;5:e7871. 10.2196/mhealth.7871 PMC5529741 28701291

[68] Phillips KM, Antoni MH, Lechner SC, Blomberg BB, Llabre MM, Avisar E, Glück S, DerHagopian R, Carver CS. Stress management intervention reduces serum cortisol and increases relaxation during treatment for nonmetastatic breast cancer. Psychosom Med. 2008;70(9):1044–1049. 18842742 10.1097/PSY.0b013e318186fb27 PMC5761725

[69] Frank R, Hargreaves R. Clinical biomarkers in drug discovery and development. Nat Rev Drug Discov. 2003;2(7):566–580. 12838269 10.1038/nrd1130

[70] Ikeda K, Ide S, Han W, Hayashida M, Uhl GR, Sora I. How individual sensitivity to opiates can be predicted by gene analyses. Trends Pharmacol Sci. 2005;26(6):311–317. 15925706 10.1016/j.tips.2005.04.001

[71] Liu R, Wang X, Aihara K, Chen L. Early diagnosis of complex diseases by molecular biomarkers, network biomarkers, and dynamical network biomarkers. Med Res Rev. 2014;34(3):455–478. 23775602 10.1002/med.21293

[72] Planchard D, Loriot Y, Goubar A, Commo F, Jean-Charles S. Differential expression of biomarkers in men and women. Semin Oncol. 2009;36(6):553–565. 19995647 10.1053/j.seminoncol.2009.09.004

[73] Fallon M, Giusti R, Aielli F, Hoskin P, Rolke R, Sharma M, Ripamonti CI. ESMO Guidelines Committee. Management of cancer pain in adult patients: ESMO clinical practice guidelines. Ann Oncol. 2018;29:iv166–iv191. 30052758 10.1093/annonc/mdy152

[74] Pons-Faudoa FP, Ballerini A, Sakamoto J, Grattoni A. Advanced implantable drug delivery technologies: Transforming the clinical landscape of therapeutics for chronic diseases. Biomed Microdevices. 2019;21(2):47. 31104136 10.1007/s10544-019-0389-6 PMC7161312

[75] Paice JA, Portenoy R, Lacchetti C, Campbell T, Cheville A, Citron M, Constine LS, Cooper A, Glare P, Keefe F, et al. Management of chronic pain in survivors of adult cancers: American Society of Clinical Oncology clinical practice guideline. J Clin Oncol. 2016;34(27):3325–3345. 27458286 10.1200/JCO.2016.68.5206

[76] Zhang SP, Lata J, Chen C, Mai J, Guo F, Tian Z, Ren L, Mao Z, Huang PH, Li P, et al. Digital acoustofluidics enables contactless and programmable liquid handling. Nat Commun. 2018;9(1):2928. 30050088 10.1038/s41467-018-05297-z PMC6062562

[77] Wheless JW, Phelps SJ. A clinician’s guide to oral extended-release drug delivery systems in epilepsy. J Pediatr Pharmacol Ther. 2018;23(4):277–292. 30181718 10.5863/1551-6776-23.4.277 PMC6117810

[78] Xu J, Tu H, Ao Z, Chen Y, Danehy R, Guo F. Acoustic disruption of tumor endothelium and on-demand drug delivery for cancer chemotherapy. Nanotechnology. 2019;30(15):154001. 30641501 10.1088/1361-6528/aafe4e

[79] Domingo-Lopez DA, Lattanzi G, H. J. Schreiber L, Wallace EJ, Wylie R, O’Sullivan J, Dolan EB, Duffy GP. Medical devices, smart drug delivery, wearables and technology for the treatment of diabetes mellitus. Adv Drug Deliv Rev. 2022;185:114280. 35405298 10.1016/j.addr.2022.114280

[80] Wen MM, el-Salamouni NS, el-Refaie WM, Hazzah HA, Ali MM, Tosi G, Farid RM, Blanco-Prieto MJ, Billa N, Hanafy AS. Nanotechnology-based drug delivery systems for Alzheimer’s disease management: Technical, industrial, and clinical challenges. J Control Release. 2017;245:95–107. 27889394 10.1016/j.jconrel.2016.11.025

[81] Wishart DS. Emerging applications of metabolomics in drug discovery and precision medicine. Nat Rev Drug Discov. 2016;15(7):473–484. 26965202 10.1038/nrd.2016.32

[82] Taylor S-S, Noor N, Urits I, Paladini A, Sadhu MS, Gibb C, Carlson T, Myrcik D, Varrassi G, Viswanath O. Complex regional pain syndrome: A comprehensive review. Pain Ther. 2021;10(2):875–892. 34165690 10.1007/s40122-021-00279-4 PMC8586273

[83] Head J, Mazza J, Sabourin V, Turpin J, Hoelscher C, Wu C, Sharan A. Waves of pain relief: a systematic review of clinical trials in spinal cord stimulation waveforms for the treatment of chronic neuropathic low back and leg pain. World Neurosurg. 2019;131:264–274.e3. 31369885 10.1016/j.wneu.2019.07.167

[84] Joosten EA, Franken G. Spinal cord stimulation in chronic neuropathic pain: Mechanisms of action, new locations, new paradigms. Pain. 2020;161(1):S104–S113. 33090743 10.1097/j.pain.0000000000001854 PMC7434213

[85] Lanza GA, Grimaldi R, Greco S, Ghio S, Sarullo F, Zuin G, De Luca A, Allegri M, Pede FD, Castagno D, et al. Spinal cord stimulation for the treatment of refractory angina pectoris: A multicenter randomized single-blind study (the SCS-ITA trial). Pain. 2011;152(1):45–52. 21084162 10.1016/j.pain.2010.08.044

[86] Tran DQ, Duong S, Bertini P, Finlayson RJ. Treatment of complex regional pain syndrome: A review of the evidence. Can J Anesth. 2010;57(2):149–166. 20054678 10.1007/s12630-009-9237-0

[87] Bendersky D, Yampolsky C. Is spinal cord stimulation safe? A review of its complications. World Neurosurg. 2014;82(6):1359–1368. 23851231 10.1016/j.wneu.2013.06.012

[88] Amann W, Berg P, Gersbach P, Gamain J, Raphael JH, Ubbink DT. Spinal cord stimulation in the treatment of non-reconstructable stable critical leg ischaemia: Results of the European Peripheral Vascular Disease Outcome Study (SCS-EPOS). Eur J Vasc Endovasc Surg. 2003;26(3):280–286. 14509891 10.1053/ejvs.2002.1876

[89] Arciola CR, Campoccia D, Montanaro L. Implant infections: Adhesion, biofilm formation and immune evasion. Nat Rev Microbiol. 2018;16(7):397–409. 29720707 10.1038/s41579-018-0019-y

[90] O’Connor AB. Neuropathic pain: Quality-of-life impact, costs and cost effectiveness of therapy. Pharmacoeconomics. 2009;27(2):95–112. 19254044 10.2165/00019053-200927020-00002

[91] Miocinovic S, Somayajula S, Chitnis S, Vitek JL. History, applications, and mechanisms of deep brain stimulation. JAMA Neurol. 2013;70(2):163–171. 23407652 10.1001/2013.jamaneurol.45

[92] Pereira EA, Green AL, Aziz TZ. Deep brain stimulation for pain. Handb Clin Neurol. 2013;116:277–294. 24112902 10.1016/B978-0-444-53497-2.00023-1

[93] Pereira EA, Aziz TZ. Neuropathic pain and deep brain stimulation. Neurotherapeutics. 2014;11(3):496–507. 24867325 10.1007/s13311-014-0278-x PMC4121442

[94] Wallace BA, Ashkan K, Benabid A-L. Deep brain stimulation for the treatment of chronic, intractable pain. Neurosurg Clin N Am. 2004;15(3):343–357. 15246342 10.1016/j.nec.2004.03.004

[95] Frizon LA, Yamamoto EA, Nagel SJ, Simonson MT, Hogue O, Machado AG. Deep brain stimulation for pain in the modern era: A systematic review. Neurosurgery. 2020;86(2):191–202. 30799493 10.1093/neuros/nyy552

[96] Kumar K, Toth C, Nath RK. Deep brain stimulation for intractable pain: A 15-year experience. Neurosurgery. 1997;40(4):736–747. 9092847 10.1097/00006123-199704000-00015

[97] Lozano AM, Lipsman N, Bergman H, Brown P, Chabardes S, Chang JW, Matthews K, McIntyre CC, Schlaepfer TE, Schulder M, et al. Deep brain stimulation: Current challenges and future directions. Nat Rev Neurol. 2019;15(3):148–160. 30683913 10.1038/s41582-018-0128-2 PMC6397644

[98] Wolkerstorfer A, Handler N, Buschmann H. New approaches to treating pain. Bioorg Med Chem Lett. 2016;26(4):1103–1119. 26774577 10.1016/j.bmcl.2015.12.103

[99] Sluka KA, Walsh D. Transcutaneous electrical nerve stimulation: Basic science mechanisms and clinical effectiveness. J Pain. 2003;4(3):109–121. 14622708 10.1054/jpai.2003.434

[100] Salman M, Kemp H, Cauldwell M, Dob D, Sutton R. Anaesthetic management of pregnant patients with cardiac implantable electronic devices: Case reports and review. Int J Obstet Anesth. 2018;33:57–66. 28899734 10.1016/j.ijoa.2017.07.011

[101] Klein MM, Treister R, Raij T, Pascual-Leone A, Park L, Nurmikko T, Lenz F, Lefaucheur JP, Lang M, Hallett M, et al. Transcranial magnetic stimulation of the brain: Guidelines for pain treatment research. Pain. 2015;156(9):1601–1614. 25919472 10.1097/j.pain.0000000000000210 PMC4545735

[102] Fregni F, Pascual-Leone A. Technology insight: Noninvasive brain stimulation in neurology—Perspectives on the therapeutic potential of rTMS and tDCS. Nat Clin Pract Neurol. 2007;3(7):383–393. 17611487 10.1038/ncpneuro0530

[103] Wassermann EM, Lisanby SH. Therapeutic application of repetitive transcranial magnetic stimulation: A review. Clin Neurophysiol. 2001;112(8):1367–1377. 11459676 10.1016/s1388-2457(01)00585-5

[104] Rostami M, Golesorkhi M, Ekhtiari H. Methodological dimensions of transcranial brain stimulation with the electrical current in human. Basic Clin Neurosci. 2013;4(3):190. 25337348 PMC4202570

[105] Yu K, Niu X, He B. Neuromodulation management of chronic neuropathic pain in the central nervous system. Adv Funct Mater. 2020;30(37):1908999. 34335132 10.1002/adfm.201908999 PMC8323399

[106] Billot M, Naiditch N, Brandet C, Lorgeoux B, Baron S, Ounajim A, Roulaud M, Roy-Moreau A, de Montgazon G, Charrier E, et al. Comparison of conventional, burst and high-frequency spinal cord stimulation on pain relief in refractory failed back surgery syndrome patients: Study protocol for a prospective randomized double-blinded cross-over trial (MULTIWAVE study). Trials. 2020;21(1):696. 32746899 10.1186/s13063-020-04587-6 PMC7397663

[107] Maloney J et al. Efficacy and safety of grass sublingual immunotherapy tablet, MK-7243: A large randomized controlled trial. Ann Allergy Asthma Immunol. 2014;112(2):146–153.e142. 24468255 10.1016/j.anai.2013.11.018

[108] Rudralingam M, Randall C, Mighell A. The use of topical steroid preparations in oral medicine in the UK. Br Dent J. 2017;223(9):633–638. 29097797 10.1038/sj.bdj.2017.880

[109] Ruffini G, Wendling F, Merlet I, Molaee-Ardekani B, Mekonnen A, Salvador R, Soria-Frisch A, Grau C, Dunne S, Miranda PC. Transcranial current brain stimulation (tCS): Models and technologies. IEEE Trans Neural Syst Rehabil Eng. 2012;21(3):333–345. 10.1109/TNSRE.2012.2200046 22949089

[110] Sengupta S, Balla VK. A review on the use of magnetic fields and ultrasound for non-invasive cancer treatment. J Adv Res. 2018;14:97–111. 30109147 10.1016/j.jare.2018.06.003 PMC6090088

[111] Papadopoulos ES, Mani R. The role of ultrasound therapy in the management of musculoskeletal soft tissue pain. Int J Low Extrem Wounds. 2020;19(4):350–358. 32856521 10.1177/1534734620948343

[112] Krishna V, Sammartino F, Rezai A. A review of the current therapies, challenges, and future directions of transcranial focused ultrasound technology: Advances in diagnosis and treatment. JAMA Neurol. 2018;75(2):246–254. 29228074 10.1001/jamaneurol.2017.3129

[113] Korbe S, Udoji EN, Ness TJ, Udoji MA. Ultrasound-guided interventional procedures for chronic pain management. Pain Manag. 2015;5(6):466–482. 10.2217/pmt.15.46 PMC4976830 26402316

[114] Lamperti M, Bodenham AR, Pittiruti M, Blaivas M, Augoustides JG, Elbarbary M, Pirotte T, Karakitsos D, LeDonne J, Doniger S, et al. International evidence-based recommendations on ultrasound-guided vascular access. Intensive Care Med. 2012;38(7):1105–1117. 22614241 10.1007/s00134-012-2597-x

[115] Werner P, al-Hamadi A, Limbrecht-Ecklundt K, Walter S, Gruss S, Traue HC. Automatic pain assessment with facial activity descriptors. IEEE Trans Affect Comput. 2016;8(3):286–299.

[116] Posada-Quintero HF, Kong Y, Chon KH. Objective pain stimulation intensity and pain sensation assessment using machine learning classification and regression based on electrodermal activity. Am J Physiol Regul Integr Comp Physiol. 2021;321(2):R186–R196. 34133246 10.1152/ajpregu.00094.2021 PMC8409909

[117] Syrowatka A, Song W, Amato MG, Foer D, Edrees H, Co Z, Kuznetsova M, Dulgarian S, Seger DL, Simona A, et al. Key use cases for artificial intelligence to reduce the frequency of adverse drug events: A scoping review. Lancet Digit Health. 2022;4(2):e137–e148. 34836823 10.1016/S2589-7500(21)00229-6

[118] Bifulco L, Anderson DR, Blankson ML, Channamsetty V, Blaz JW, Nguyen-Louie TT, Scholle SH. Evaluation of a chronic pain screening program implemented in primary care. JAMA Netw Open. 2021;4(7):e2118495. 34313738 10.1001/jamanetworkopen.2021.18495 PMC8317006

[119] Nielsen CS, Stubhaug A, Price DD, Vassend O, Czajkowski N, Harris JR. Individual differences in pain sensitivity: Genetic and environmental contributions. Pain. 2008;136(1-2):21–29. 17692462 10.1016/j.pain.2007.06.008

[120] Bates MS, Edwards WT, Anderson KO. Ethnocultural influences on variation in chronic pain perception. Pain. 1993;52(1):101–112. 8446431 10.1016/0304-3959(93)90120-E

[121] Yan T, Zhang G, Chai H, Qu L, Zhang X. Flexible biosensors based on colorimetry, fluorescence, and electrochemistry for point-of-care testing. Front Bioeng Biotechnol. 2021;9:753692. 34650963 10.3389/fbioe.2021.753692 PMC8505690

[122] Fraser AG, Daubert JC, van de Werf F, Estes NAM, Smith SC, Krucoff MW, Vardas PE, Komajda M, on behalf of the participants, Anker S, et al. Clinical evaluation of cardiovascular devices: Principles, problems, and proposals for European regulatory reform: Report of a policy conference of the European Society of Cardiology. Eur Heart J. 2011;32(13):1673–1686. 21572115 10.1093/eurheartj/ehr171

[123] Dinesen B, Nonnecke B, Lindeman D, Toft E, Kidholm K, Jethwani K, Young HM, Spindler H, Oestergaard CU, Southard JA, et al. Personalized telehealth in the future: A global research agenda. J Med Internet Res. 2016;18(3):e53. 26932229 10.2196/jmir.5257 PMC4795318

[124] Jayawardana S, Forman R, Johnston-Webber C, Campbell A, Berterame S, de Joncheere C, Aitken M, Mossialos E. Global consumption of prescription opioid analgesics between 2009-2019: A country-level observational study. EClinicalMedicine (2021);42:101198. 34820610 10.1016/j.eclinm.2021.101198 PMC8599097

[125] Liu JX, Goryakin Y, Maeda A, Bruckner T, Scheffler R. Global health workforce labor market projections for 2030. Hum Resour Health. 2017;15(1):11. 28159017 10.1186/s12960-017-0187-2 PMC5291995

[126] Qin A, Qin W, Hu F, Wang M, Yang H, Li L, Chen C, Bao B, Xin T, Xu L. Does unequal economic development contribute to the inequitable distribution of healthcare resources? Evidence from China spanning 2001–2020. Glob Health. 2024;20(1):20. 10.1186/s12992-024-01025-z PMC10913684 38443966

[127] Glasziou P, Straus S, Brownlee S, Trevena L, Dans L, Guyatt G, Elshaug AG, Janett R, Saini V. Evidence for underuse of effective medical services around the world. Lancet. 2017;390(10090):169–177. 28077232 10.1016/S0140-6736(16)30946-1

[128] Varshney P, Simmhan Y. Demystifying fog computing: Characterizing architectures, applications and abstractions. In: *2017 IEEE 1st International Conference on Fog and Edge Computing (ICFEC)*. Madrid, Spain, IEEE; 2017. p. 115–124.

[129] Cerdá M, Krawczyk N, Hamilton L, Rudolph KE, Friedman SR, Keyes KM. A critical review of the social and behavioral contributions to the overdose epidemic. Annu Rev Public Health. 2021;42:95–114. 33256535 10.1146/annurev-publhealth-090419-102727 PMC8675278

[130] Boun SS, Omonaiye O, Yaya S. Prevalence and health consequences of nonmedical use of tramadol in Africa: A systematic scoping review. PLOS Global Public Health. 2024;4:e0002784. 38236813 10.1371/journal.pgph.0002784 PMC10796000

[131] Zacny J, Bigelow G, Compton P, Foley K, Iguchi M, Sannerud C. College on problems of drug dependence taskforce on prescription opioid non-medical use and abuse: Position statement. Drug Alcohol Depend. 2003;69(3):215–232. 12633908 10.1016/s0376-8716(03)00003-6

[132] Turk DC, Wilson HD, Cahana A. Treatment of chronic non-cancer pain. Lancet. 2011;377(9784):2226–2235. 21704872 10.1016/S0140-6736(11)60402-9

[133] Eisenberg DM, Kessler RC, van Rompay MI, Kaptchuk TJ, Wilkey SA, Appel S, Davis RB. Perceptions about complementary therapies relative to conventional therapies among adults who use both: Results from a national survey. Ann Intern Med. 2001;135(3):344–351. 11529698 10.7326/0003-4819-135-5-200109040-00011

[134] Nandakumar R, Gollakota S, Sunshine JE. Opioid overdose detection using smartphones. Sci Transl Med. 2019;11(474):eaau8914. 30626717 10.1126/scitranslmed.aau8914

[135] Block BM, Liu SS, Rowlingson AJ, Cowan AR, Cowan JA Jr, Wu CL. Efficacy of postoperative epidural analgesia: A meta-analysis. JAMA. 2003;290(18):2455–2463. 14612482 10.1001/jama.290.18.2455

[136] Korsch BM, Gozzi EK, Francis V. Gaps in doctor-patient communication: I Doctor-patient interaction and patient satisfaction. Pediatrics. 1968;42(5):855–871. 5685370

[137] Zhang M, Zhu L, Lin SY, Herr K, Chi CL, Demir I, Dunn Lopez K, Chi NC. Using artificial intelligence to improve pain assessment and pain management: A scoping review. J Am Med Inform Assoc. 2023;30(3):570–587. 36458955 10.1093/jamia/ocac231 PMC9933069

[138] Johnson KB, Wei WQ, Weeraratne D, Frisse ME, Misulis K, Rhee K, Zhao J, Snowdon JL. Precision medicine, AI, and the future of personalized health care. Clin Transl Sci. 2021;14(1):86–93. 32961010 10.1111/cts.12884 PMC7877825

[139] Cai H, Ao Z, Tian C, Wu Z, Liu H, Tchieu J, Gu M, Mackie K, Guo F. Brain organoid reservoir computing for artificial intelligence. Nat Electron. 2023;6:1032–1039.

[140] Leroux A, Rzasa-Lynn R, Crainiceanu C, Sharma T. Wearable devices: Current status and opportunities in pain assessment and management. Digit Biomark. 2021;5(1):89–102. 34056519 10.1159/000515576 PMC8138140

[141] Ahmed MU, Barua S, Begum S. Artificial intelligence, machine learning and reasoning in health informatics—Case studies. *Signal Process Tech Comput Health Inform*. 2021:261–291.

[142] Guo F, Li P, French JB, Mao Z, Zhao H, Li S, Nama N, Fick JR, Benkovic SJ, Huang TJ. Controlling cell–cell interactions using surface acoustic waves. Proc Natl Acad Sci USA. 2015;112(1):43–48. 25535339 10.1073/pnas.1422068112 PMC4291613

[143] Wu Z, Cai H, Tian C, Ao Z, Jiang L, Guo F. Exploiting sound for emerging applications of extracellular vesicles. Nano Res. 2024;17(2):462–475. 38712329 10.1007/s12274-023-5840-6 PMC11073796

[144] Cai H, Ao Z, Wu Z, Nunez A, Jiang L, Carpenter RL, Nephew KP, Guo F. Profiling cell–matrix adhesion using digitalized acoustic streaming. Anal Chem. 2019;92(2):2283–2290. 10.1021/acs.analchem.9b05065 31880433

[145] Zhu Q, Huang L, Yang Q, Ao Z, Yang R, Krzesniak J, Lou D, Hu L, Dai X, Guo F, et al. Metabolomic analysis of exosomal-markers in esophageal squamous cell carcinoma. Nanoscale. 2021;13(39):16457–16464. 34648610 10.1039/d1nr04015d

[146] Guo F, Xie Y, Li S, Lata J, Ren L, Mao Z, Ren B, Wu M, Ozcelik A, Huang TJ. Reusable acoustic tweezers for disposable devices. Lab Chip. 2015;15(24):4517–4523. 26507411 10.1039/c5lc01049g PMC4683015

[147] Xu J, Danehy R, Cai H, Ao Z, Pu M, Nusawardhana A, Rowe-Magnus D, Guo F. Microneedle patch-mediated treatment of bacterial biofilms. ACS Appl Mater Interfaces. 2019;11(16):14640–14646. 30933463 10.1021/acsami.9b02578

[148] Wu Z, Cai H, Ao Z, Nunez A, Liu H, Bondesson M, Guo S, Guo F. A digital acoustofluidic pump powered by localized fluid-substrate interactions. Anal Chem. 2019;91(11):7097–7103. 31083981 10.1021/acs.analchem.9b00069

[149] Guo F, Mao Z, Chen Y, Xie Z, Lata JP, Li P, Ren L, Liu J, Yang J, Dao M, et al. Three-dimensional manipulation of single cells using surface acoustic waves. Proc Natl Acad Sci USA. 2016;113(6):1522–1527. 26811444 10.1073/pnas.1524813113 PMC4760790

[150] Xing Y, Cheng H, Yang C, Xiao Z, Yan C, Chen FF, Li J, Zhang Y, Cui C, Li J, et al. Evaluation of skin sympathetic nervous activity for classification of intracerebral hemorrhage and outcome prediction. Comput Biol Med. 2023;166:107397. 37804780 10.1016/j.compbiomed.2023.107397

[151] Yang J, Yang J, Gong X, Zheng Y, Yi S, Cheng Y, Li Y, Liu B, Xie X, Yi C, et al. Recent progress in microneedles-mediated diagnosis, therapy, and theranostic systems. Adv Healthc Mater. 2022;11:2102547. 10.1002/adhm.202102547 35034429

[152] Colloca L, Raghuraman N, Wang Y, Akintola T, Brawn-Cinani B, Colloca GC, Kier C, Varshney A, Murthi S. Virtual reality: Physiological and behavioral mechanisms to increase individual pain tolerance limits. Pain. 2020;161(9):2010–2021. 32345915 10.1097/j.pain.0000000000001900 PMC7584744

[153] Torous J, Bucci S, Bell IH, Kessing LV, Faurholt-Jepsen M, Whelan P, Carvalho AF, Keshavan M, Linardon J, Firth J. The growing field of digital psychiatry: Current evidence and the future of apps, social media, chatbots, and virtual reality. World Psychiatry. 2021;20(3):318–335. 34505369 10.1002/wps.20883 PMC8429349

[154] Moreau S, Thérond A, Cerda IH, Studer K, Pan A, Tharpe J, Crowther JE, Abd-Elsayed A, Gilligan C, Tolba R, et al. Virtual reality in acute and chronic pain medicine: An updated review. Curr Pain Headache Rep. 2024. 10.1007/s11916-024-01246-2 38587725

[155] Teutsch C. Patient–doctor communication. Medical Clin North Am. 2003;87(5):1115–1145. 10.1016/s0025-7125(03)00066-x 14621334

[156] Robinson A. Rationale for cost-effective laboratory medicine. Clin Microbiol Rev. 1994;7(2):185–199. 8055467 10.1128/cmr.7.2.185 PMC358317

[157] Fotheringham D, Wiles MA. The effect of implementing chatbot customer service on stock returns: An event study analysis. J Acad Mark Sci. 2023;51:802–822.

[158] Prochaska JJ, Vogel EA, Chieng A, Kendra M, Baiocchi M, Pajarito S, Robinson A. A therapeutic relational agent for reducing problematic substance use (Woebot): Development and usability study. J Med Internet Res. 2021;23:e24850. 33755028 10.2196/24850 PMC8074987

[159] Reis F, Lenz C. Performance of artificial intelligence (AI)-powered chatbots in the assessment of medical case reports: Qualitative insights from simulated scenarios. Cureus. 2024;16(2):e53899. 38465163 10.7759/cureus.53899 PMC10925004

[160] Aminizadeh S, Heidari A, Dehghan M, Toumaj S, Rezaei M, Navimipour NJ, Stroppa F, Unal M. Opportunities and challenges of artificial intelligence and distributed systems to improve the quality of healthcare service. Artif Intell Med. 2024;149:102779. 38462281 10.1016/j.artmed.2024.102779

[161] Kumar N. *WHO normative guidelines on pain management*. Geneva: World Health Organization; 2007. p. 3–4.

[162] Gelband H, Sankaranarayanan R, Gauvreau CL, Horton S, Anderson BO, Bray F, Cleary J, Dare AJ, Denny L, Gospodarowicz MK, et al. Costs, affordability, and feasibility of an essential package of cancer control interventions in low-income and middle-income countries: Key messages from Disease Control Priorities. Lancet. 2016;387:2133–2144. 26578033 10.1016/S0140-6736(15)00755-2

[163] Lee D, Yoon SN. Application of artificial intelligence-based technologies in the healthcare industry: Opportunities and challenges. Int J Environ Res Public Health. 2021;18(1):271. 33401373 10.3390/ijerph18010271 PMC7795119

[164] Yang S, Slotcavage D, Mai JD, Guo F, Li S, Zhao Y, Lei Y, Cameron CE, Huang TJ. Electrochemically created highly surface roughened Ag nanoplate arrays for SERS biosensing applications. J Mater Chem C. 2014;2(39):8350–8356. 10.1039/C4TC01276C PMC4217216 25383191

[165] Wu J, Li Q, Fan L, Lan Z, Li P, Lin J, Hao S. High-performance polypyrrole nanoparticles counter electrode for dye-sensitized solar cells. J Power Sources. 2008;181(1):172–176.

[166] Liu X-M, Huang Z, Oh S, Zhang B, Ma PC, Yuen MMF, Kim JK. Carbon nanotube (CNT)-based composites as electrode material for rechargeable Li-ion batteries: A review. Compos Sci Technol. 2012;72(2):121–144.

[167] Saxena P, Shukla P. A comprehensive review on fundamental properties and applications of poly (vinylidene fluoride) (PVDF). Adv Compos Hybrid Mater. 2021;4:8–26.

[168] Cafarelli A, Marino A, Vannozzi L, Puigmartí-Luis J, Pané S, Ciofani G, Ricotti L. Piezoelectric nanomaterials activated by ultrasound: The pathway from discovery to future clinical adoption. ACS Nano. 2021;15(1):11066–11086. 34251189 10.1021/acsnano.1c03087 PMC8397402

[169] Ryu G-S, You J, Kostianovskii V, Lee EB, Kim Y, Park C, Noh YY. Flexible and printed PPG sensors for estimation of drowsiness. IEEE Trans Electron Devices. 2018;65(7):2997–3004.

[170] Xu H, Li J, Leung BHK, Poon CCY, Ong BS, Zhang Y, Zhao N. A high-sensitivity near-infrared phototransistor based on an organic bulk heterojunction. Nanoscale. 2013;5(23):11850–11855. 24126789 10.1039/c3nr03989g

[171] Belser RB, Hicklin WH. Temperature coefficients of resistance of metallic films in the temperature range 25 to 600 C. J Appl Phys. 1959;30(3):313–322.

